# CD8^+^*γδ* T Cells Are More Frequent in CMV Seropositive Bone Marrow Grafts and Display Phenotype of an Adaptive Immune Response

**DOI:** 10.1155/2019/6348060

**Published:** 2019-12-06

**Authors:** Ahmed Gaballa, Lucas C. M. Arruda, Emelie Rådestad, Michael Uhlin

**Affiliations:** ^1^Department of Clinical Science, Intervention and Technology, Karolinska Institutet, Stockholm, Sweden; ^2^Department of Applied Physics, Science for Life Laboratory, Royal Institute of Technology, Stockholm, Sweden; ^3^Department of Immunology and Transfusion Medicine, Karolinska University Hospital, Stockholm, Sweden

## Abstract

The role of gamma delta (*γδ*) T cells in human cytomegalovirus (HCMV) immune surveillance has been the focus of research interest for years. Recent reports have shown a substantial clonal proliferation of *γδ* T cells in response to HCMV, shedding light on the adaptive immune response of *γδ* T cells. Nevertheless, most efforts have focused on V*δ*2^neg^*γδ* T cell subset while less attention has been given to investigate other less common *γδ* T cell subsets. In this regard, a distinct subpopulation of *γδ* T cells that expresses the CD8 coreceptor (CD8^+^*γδ* T cells) has not been thoroughly explored. Whether it is implicated in HCMV response and its ability to generate adaptive response has not been thoroughly investigated. In this study, we combined flow cytometry and immune sequencing of the TCR *γ*-chain (*TRG*) to analyze in-depth bone marrow (BM) graft *γδ* T cells from CMV seropositive (CMV+) and CMV seronegative (CMV-) donors. We showed that the frequency of CD8^+^*γδ* T cells was significantly higher in CMV+ grafts compared to CMV- grafts (*P* < 0.001). Further characterization revealed that CD8^+^*γδ* T cells from CMV+ grafts express V*γ*9^−^ and preferentially differentiated from a naive to terminal effector memory phenotype (CD27^low/-^CD45RO^−^). In line with these findings, *TRG* immune sequencing revealed clonal focusing and reduced usage of the V*γ*9/JP gene segment in a CMV+ graft. Furthermore, CD8^+^*γδ* T cells showed an enhanced response to TCR/CD3 and cytokine stimulation in contrast to CD8^−^*γδ* T cells. We conclude that *γδ* T cells in BM grafts are reshaped by donor CMV serostatus and highlight the potential adaptive role of CD8^+^*γδ* T cells in HCMV immune response.

## 1. Introduction

Human cytomegalovirus (HCMV) is a DNA virus that belongs to the *β*-herpes virus family [1]. In immunocompetent individuals, HCMV establishes a lifelong latent infection that is usually asymptomatic. However, in conditions where the immune system is dampened, such following allogeneic Hematopoietic Cell Transplantation (HCT), HCMV can be life-threatening, rendering CMV infection/reactivation a major cause of morbidity and mortality after HCT [2].

Human *γδ* T cells are unconventional T cells that express a T cell antigen receptor (TCR) formed by *γ* and *δ* chains and fundamentally differ from *αβ* T cells in their major histocompatibility complex- (MHC-) independent antigen recognition [3]. In allogeneic HCT, *γδ* T cell reconstitution occurs shortly after transplantation [4], a process that has been associated with a favorable outcome, indicating their crucial role in protection against tumors and pathogens [5–7].

The role of *γδ* T cells in HCMV immune surveillance has been shown previously [8]. However, the underlying immune mechanism and the ligand/s mediating *γδ* T cell activation are poorly understood [8, 9]. Furthermore, whether *γδ* T cells respond to HCMV through innate or adaptive immune pathways is unclear. V*γ*9^+^V*δ*2^+^ cells express a semi-invariant TCR and respond to a limited range of nonpeptide antigens such as phosphoantigens, rendering their response innate-like in nature. In contrast, V*δ*2^neg^*γδ* T cells have a wider range of ligands and display high diverse TCR repertoire at birth that become focused at adulthood, sharing more properties of adaptive immunity [10, 11].

HCMV infection is associated with a remarkable proliferation of V*δ*2^neg^*γδ* subsets, particularly V*δ*1^+^ cells [1]. Recently, next-generation sequencing of the TCR chains *δ* (*TRD*) and *γ* (*TRG)* has allowed an in-depth analysis of the *γδ* TCR repertoire reshaping in response to HCMV. Using this state-of-the-art technique, Ravens et al. and Davey et al. have revealed for the first time CMV-associated clonotypic changes in the *γδ* TCR repertoire [12–14]; their reports provide strong evidence for the ability of *γδ* T cells to mount a virus-specific nonconventional adaptive immune response.

The majority of human adults circulating *γδ* T cells are double negative for CD8 and CD4 coreceptors (CD4^−^ CD8^−^), partially accounting for their MHC independence [13]. However, a small subpopulation of *γδ* T cells expresses the CD8 coreceptor (CD8^+^*γδ* T cells). Reports from several research groups including ours suggested distinct immunobiology of this subset [15, 16]. In the context of allogenic HCT, the role of this subset in HCMV infection has not yet been fully described. Whether CD8^+^*γδ* T cells undergo clonal proliferation in response to HCMV and if they are capable of mounting adaptive function has so far not been shown. It is therefore fundamental to address their potential role in CMV immune response. In this study, we characterized *γδ* T cells in BM grafts from CMV+ and CMV- donors using multicolor flow cytometry in addition to immune sequencing of the TCR *γ* chain (TRG).

## 2. Subjects and Methods

### 2.1. Donor Characteristics and Ethical Approval

A total of 16 samples (13 males and 3 females) were obtained from BM grafts before allogeneic HCT at the Cell Therapy and Allogeneic Stem Cell Transplantation (CAST), Karolinska University Hospital, Sweden. Out of 16 donors, 7 were CMV seropositive (CMV+) and 9 were CMV seronegative (CMV-). The median age of the donors was 28 and 22 years for CMV+ and CMV- donors, respectively. Written informed consent for sample collection and subsequent analysis was provided. The study was approved by the regional ethical review board in Stockholm (2008/206-31, 2010/760-31/1, 2013/2215-32, and 2017/469-32).

### 2.2. Sample Preparation

Mononuclear cells (MNC) were freshly isolated from BM grafts by density gradient centrifugation (Lymphoprep, Fresenius Kabi, Oslo, Norway) as described previously [17], were cryopreserved in RPMI-1640 media containing 10% DMSO and supplemented with 10% human AB serum, and were stored in liquid nitrogen freezer until time of analysis.

### 2.3. Multicolor Flow Cytometry

**Figure 1:**
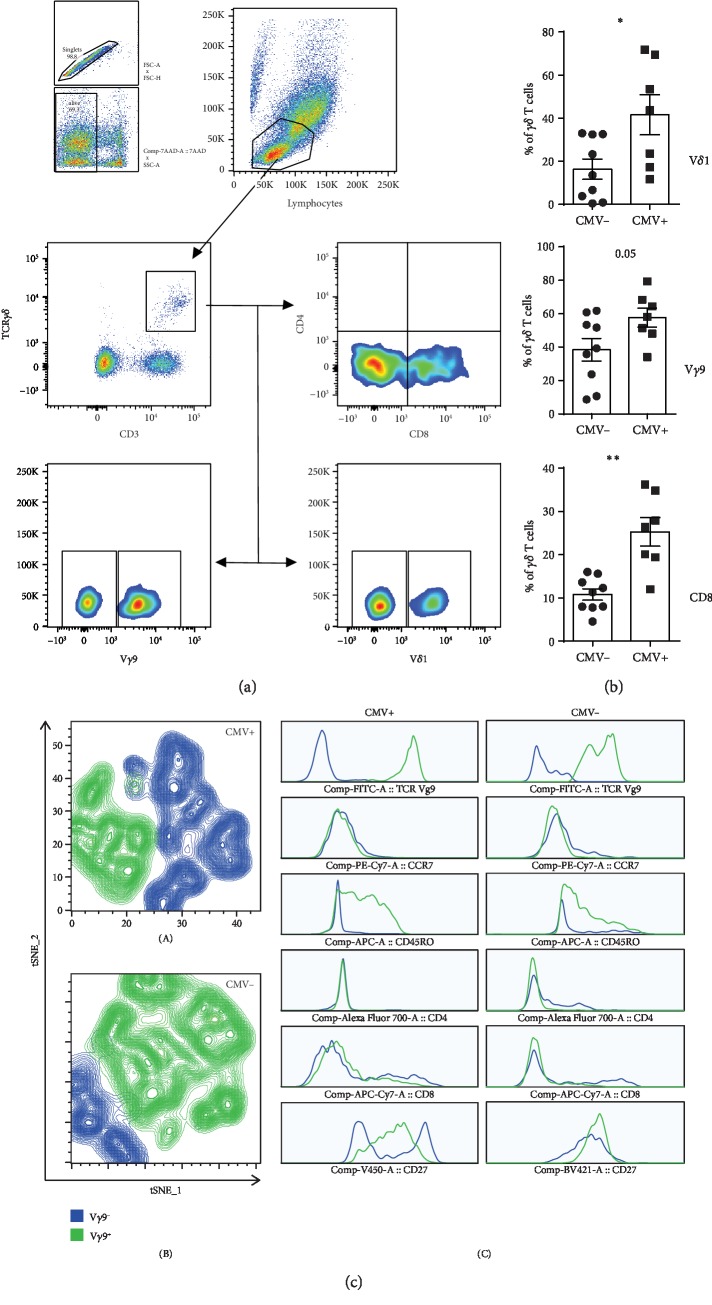
Characterization of *γδ* T cells in BM grafts. (a) Representative FACS plot showing gating strategy for different *γδ* T cell subsets; (b) proportions of V*δ*1+, V*γ*9^−^, and CD8^+^*γδ* T cells within CMV+ and CMV- grafts; (c) dimensionally reduced plots (tSNE) of *γδ* T cells in CMV+ (A) and CMV- (B) and tSNE generated histograms (C) from CMV+ and CMV- grafts. V*γ*9^−^ subsets are indicated in blue while V*γ*9^+^ subsets are indicated in green color.

Cryopreserved samples were thawed, washed, and resuspended in PBS. Surface staining was performed according to standard protocols as published before [18]. Immunophenotyping was performed using fluorochrome-conjugated anti-human monoclonal antibodies (mAb) as follows: CD3-BV450 (UCHT1), CD3-BV510 (UCHT1), CD4-Alexa Fluor 700 (RPA-T4), CD8-APC-Cy7 (SK1), CD27-BV421 (M-T271), CD45RO-APC (UCHL1), CD197 (CCR-7)-PE-Cy7 (3D12), and CD69-FITC (L78) (BD Biosciences); CD158b-PE-Cy7 (DX27) and TCR V*γ*9-FITC (B3) (BioLegend); TCR V*δ*1-FITC (TS8.2) (Thermo Scientific); and TCR pan *γδ*-PE (REA591) and TIM3-APC (F38-2E2) (Miltenyi Biotec). FACS CANTO (BD Biosciences, San Jose, CA, USA) was used to acquire samples, and FlowJo V10 (TreeStar) was used to analyze the results. The gating strategy is shown in Figure 1(a).

Manual gating was used to characterize individual samples, and subsequently, data were downsampled and merged (concatenated) for further visualization using dimensionality reduction algorithm plugin, t-Distributed Stochastic Neighbor Embedding (tSNE).

### 2.4. *γδ* Genomic DNA Extraction and Immunosequencing

MNCs from one CMV+ and one CMV- BM grafts were thawed, *γδ* T cells were sorted using the TCR *γ*/*δ* T cell isolation kit (Miltenyi Biotec) according to the manufacturer's protocol, and *γδ* purity was confirmed by FACS. Next, genomic DNA was extracted using the EZ1® DNA Blood Kit and EZ1 instruments (Qiagen, Germany) according to the manufacturer's instructions. Concentration and purity of eluted DNA were analyzed using NanoDrop 2000 (Thermo Fisher Scientific), and DNA samples were stored at -20°C. An average of 1 *μ*g of genomic DNA was used for high throughput sequencing of the CDR3 region of *TRG* using the ImmunoSEQ platform (Adaptive Biotechnologies, Seattle, WA) as described previously [19]. Briefly, amplification of V-J segments was performed in a bias-controlled multiplex PCR reaction using primers specific for V*γ* and J*γ* gene segments. A specific algorithm was then applied to correct for sequencing error. CDR3 segments were annotated according to the International ImmunoGeneTics collaboration.

### 2.5. TCR *γδ* CDR3 Spectratyping

CD8^+^ and CD8^−^*γδ* T cells were sorted from CMV+ grafts on a cell sorter (Sony MA900, Sony Biotechnology Inc.). RNA was extracted (AllPrep DNA/RNA Mini Kit, Qiagen, Germany) and immediately converted to cDNA (SuperScript™ IV VILO™ Master Mix, Thermo Fisher Scientific) as previously described [17]. The CDR3 region for V*γ*2, V*γ*3, V*γ*4, V*γ*5, V*γ*9, and V*δ*1 was amplified by PCR using forward primers specific for each V gene segment and a common 5′FAM-labeled reverse primer for the constant *γ* (C*γ*) or *δ* (C*δ*) genes as described elsewhere [20] ([Supplementary-material supplementary-material-1]). PCR conditions and spectratyping were performed according to a protocol described in detail previously [16].

### 2.6. T Cell Culture and Proliferation Assays

CD3^+^ T cells from donor BM grafts were magnetically bead sorted (Pan T Cell Isolation Kit, Miltenyi Biotec) and labeled using CellTrace violet (Thermo Fisher Scientific) according to the manufacturer's instructions. Labeled T cells were cultured in a 96-well plate (1 × 10^6^ cells/mL) in a complete RPMI-1640 medium (containing 10% human AB serum, 50 *μ*g/mL penicillin/streptomycin) either alone (unstimulated) or in the presence of anti-CD3 (clone OKT3, BioLegend), IL-7, IL-15, or IL-18 at 30 ng/mL (PeproTech) and were incubated at 37°C and 5% CO_2_ for 5 or 7 days (for anti-CD3 or cytokines, respectively). Cells were analyzed by FACS, and proliferating cells were defined as % of CellTrace violet (CTV) low cells compared to unstimulated conditions. In addition to proliferation assay, staining for activation/exhaustion surface markers (CD69, TIM3, and KIR2DL2/3) was performed.

### 2.7. Bioinformatics and Statistics

Parametric test statistics were used throughout the study after confirming that assumptions of normality were not violated using the Shapiro test and Q-Q plots. When comparing two groups, the Student *t*-test or paired *t*-test was used as indicated. ANOVA followed by post hoc multiple comparisons (Tukey's correction) were used when three or more unrelated groups were compared, and repeated measures ANOVA when the compared groups were related (paired). A *P* value < 0.05 was considered statistically significant, and the following significance levels were used: ^∗^*P* < 0.05, ^∗∗^*P* < 0.01, and ^∗∗∗^*P* < 0.001. GraphPad Prism version 6.00 for Windows (GraphPad Software, La Jolla, California, USA) and IBM SPSS Statistics for Windows, Version 24.0. (Armonk, NY: IBM Corp.) were used to perform statistics. The ImmunoSEQ tool was used for initial handling of sequencing data, and diversity, clonal space homeostasis, V-J segment usage, CDR3 spectratyping, and repertoire overlap were performed using specific packages as previously described [19].

## 3. Results

### 3.1. Characterization of *γδ* T Cell Subsets in BM Grafts

To address whether *γδ* T cell proportions in BM grafts are influenced by donor CMV serostatus, we characterized *γδ* T cells from CMV+ (*n* = 7) and CMV- (*n* = 9) BM grafts using a multicolor flow cytometer (Figure 1(a)). Immunophenotyping results showed no significant difference in the frequency of total *γδ* T cells between CMV+ and CMV- grafts (data not shown). However, further analysis of *γδ* T cell subsets revealed increased proportions of V*δ*1^+^ and V*γ*9^−^ subsets in CMV+ compared to CMV- BM grafts (mean frequency = 41.5% vs. 16.3%, *P* = 0.02 and 57.6% vs. 38.3%, *P* = 0.05, respectively) (Figure 1(b)). Strikingly, the frequency of CD8^+^*γδ* T cells was significantly higher in CMV+ grafts as compared to CMV- grafts (mean frequency = 25.2% vs. 10.7%, *P* < 0.001).

To gain more insight, we used a dimensionality reduction algorithm (tSNE) to visualize clusters of V*γ*9^+^ and V*γ*9^−^ subpopulations from CMV+ and CMV- grafts. V*γ*9^−^ subset represented a predominant distinct cluster of *γδ* T cells within CMV+ grafts compared to CMV- grafts (Figure 1(c)). Furthermore, tSNE-generated histograms from V*γ*9^−^ and V*γ*9^+^ subpopulations showed remarkable downregulation of CD45RO in V*γ*9^−^ subpopulation when compared to V*γ*9^+^ both in CMV+ and in CMV- grafts. Additionally, CD27 downregulation was more prominent in V*γ*9^−^ subpopulation in CMV+ grafts (Figure 1(c)).

### 3.2. V*γ*9^−^ Subsets within CMV+ Grafts Are Differentiated towards a Terminal Effector Phenotype

**Figure 2:**
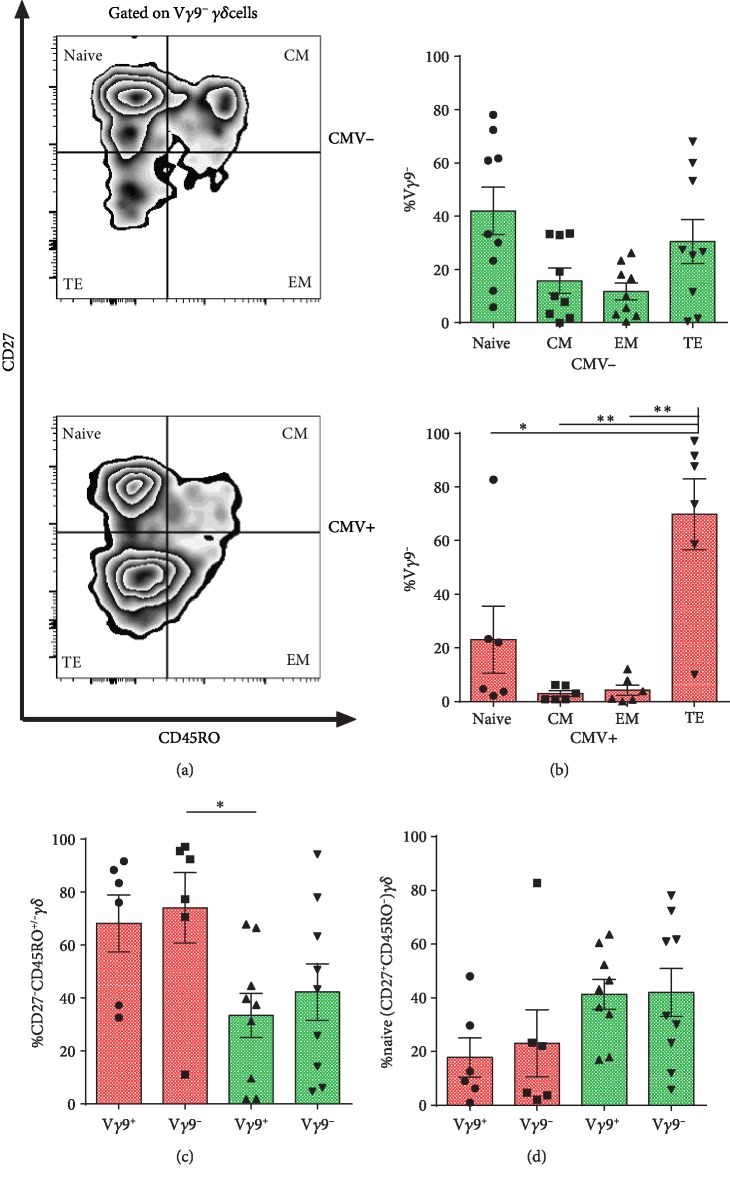
V*γ*9^−^ subsets within CMV+ grafts are enriched with an effector phenotype. (a) A representative FACS plot showing memory phenotype in V*γ*9^−^ subsets of CMV- and CMV+ grafts. (b) Proportions of naïve (CD27^+^CD45RO^−^), CM (CD27^+^CD45RO^+^), EM (CD27low/^−^CD45RO^+^), TE (CD27low/^−^CD45RO^−^) *γδ* T cells among V*γ*9^−^ subset of CMV- grafts and CMV+ grafts. Repeated measures ANOVA is used. Proportions of effector (CD27low/^−^CD45RO^−^/^+^) *γδ* T cells (c) and naïve *γδ* T cells (d) in V*γ*9^−^ and V*γ*9^+^ subsets in CMV+ and CMV- grafts. ANOVA is used. Bar and whiskers represent the mean and S.E.M.

The ability to differentiate from naïve to a memory phenotype is a characteristic of the adaptive immunity. To address this, we analyzed the frequency of naive CD27^+^CD45RO^−^, central memory (CM) CD27^+^CD45RO^+^, effector memory (EM) CD27^low/-^CD45RO^+^, and terminal effector (TE) CD27^low/-^CD45RO^−^ phenotypes among V*γ*9 subsets in CMV+ and CMV- grafts (Figures 2(a) and 2(b)). Proportions of CD27^low/-^CD45RO^−^ (TE) V*γ*9^−^*γδ* T cells were markedly increased in CMV+ grafts, whereas no difference was found in CMV- grafts (Figure 2(b)). Additionally, V*γ*9^−^*γδ* T cells from CMV+ grafts displayed a higher frequency of effector phenotype, CD27^low/-^CD45RO^-/+^ (combined EM and TE) *γδ* T cells (Figure 2(c)), and lower frequency of naïve phenotype though it did not reach the significant level (Figure 2(d)).

### 3.3. CD8^+^*γδ* T Cells within CMV+ BM Grafts Express V*γ*9^−^ and Preferentially Display Effector Phenotype

As CMV+ grafts showed significantly increased proportions of CD8^+^*γδ* T cells, we sought to further characterize this subset. Interestingly, comparison between CD8^+^ and CD8^−^*γδ* T cells revealed increased proportions of V*γ*9^−^ in CD8^+^*γδ* T cells from CMV+ grafts compared to both CD8^+^ and CD8^−^*γδ* T cells from CMV- grafts ([Supplementary-material supplementary-material-1]).

**Figure 3:**
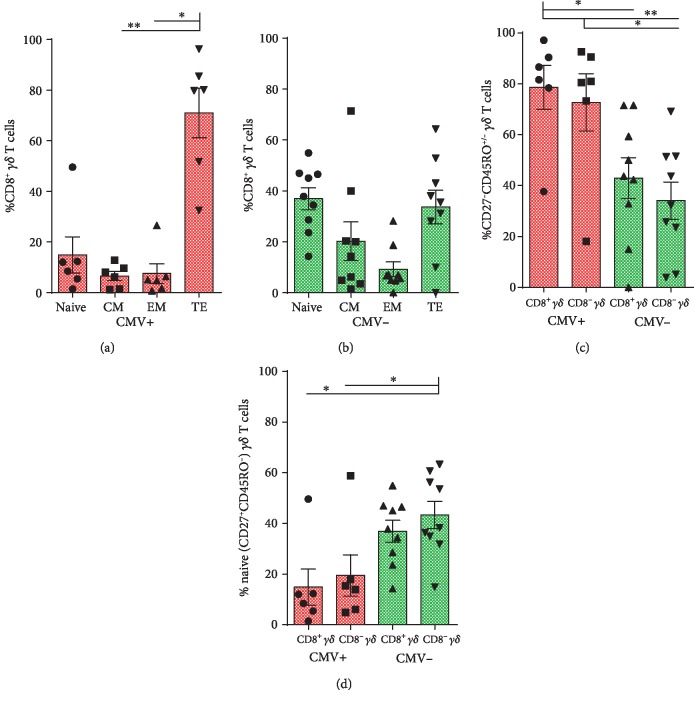
Characterization of CD8^+^*γδ* T cells within BM grafts. Proportions of naïve (CD27^+^CD45RO^−^), CM (CD27^+^CD45RO^+^), EM (CD27^low/-^CD45RO^+^), TE (CD27^low/-^CD45RO^−^) *γδ* T cells among CD8^+^*γδ* subset of CMV+ (a) and CMV- (b) BM grafts. Repeated measures ANOVA is used. Proportions of effector (CD27^low/-^CD45RO^-/+^) *γδ* T cells (c) and naïve *γδ* T cells (d) in CD8^+^*γδ* and CD8^−^*γδ* subsets in CMV+ and CMV- grafts. ANOVA is used. Bar and whiskers represent the mean and S.E.M.

Next, we investigated whether the increased frequency of CD8^+^*γδ* T cells is linked to differentiation. Comparing the frequency of different memory phenotypes revealed increased proportions of CD27^low/-^CD45RO^−^ (TE) *γδ* T cells among CD8^+^*γδ* T cells only in CMV+ grafts (Figures 3(a) and 3(b)). Importantly, the frequency of combined EM and TE phenotypes (CD27^low/-^CD45RO^−^/^+^) was higher among CD8^+^*γδ* T cells from CMV+ grafts as compared to either CD8^+^ or CD8^−^*γδ* T cells from CMV- grafts (Figure 3(c)). Consistently, CD8^+^*γδ* T cells from CMV+ grafts showed decreased proportions of naïve *γδ* T cells compared to CD8^+^ or CD8^−^*γδ* T cells from CMV- grafts (Figure 3(d)). In line with this memory phenotype, *γδ* T cells from CMV- grafts tend to express more CCR7^+^ when compared to CMV+ grafts ([Supplementary-material supplementary-material-1]).

### 3.4. *γδ* TRG Repertoire is Clonally Focused in CMV+ Grafts

**Figure 4:**
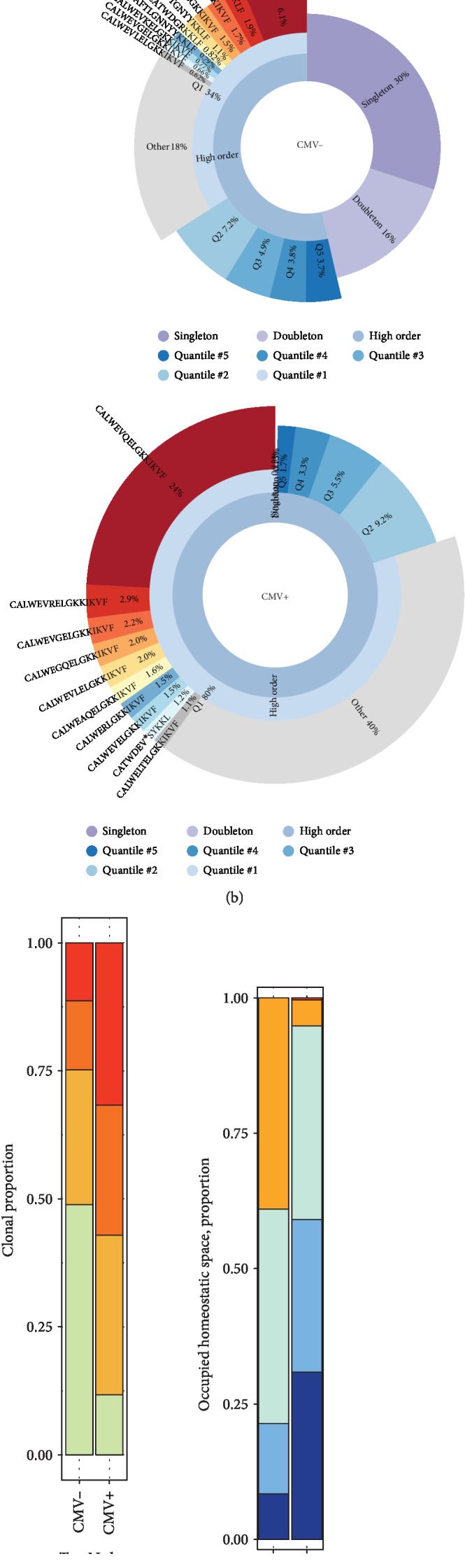
CMV+ BM grafts present less TRG diversity and high clonality. (a) Tree plots showing a CMV- and CMV+ BM graft donor TRG repertoire. Each CDR3 clonotype is colored accordingly to its amino acid sequence and is sized in relation to its repertoire frequency (the colors were chosen randomly and does not match between plots). (b) Quantile plots of a CMV- and CMV+ BM graft depicting the top 10 most frequent clonotypes. The pie chart is divided into singletons (clonotypes represented by a single read), doubletons (two reads), and high-order clonotypes (three and more reads). High-order clonotypes are divided into five quantiles (top 20% of unique high-order clonotypes and so on). The size of each segment is the cumulative frequency of all clonotypes that fall into the corresponding frequency category. (c) The clonal proportion of the top *n* clonotypes. Red bars represent the TRG proportion taken by the 10 most abundant clones shown in (b). (d) Proportion of homeostatic space occupied by clonotypes classified as hyperexpanded (0.01–1), large (0.001–0.01), medium (0.0001–0.001), small (0.00001–0.0001), and rare (0–0.00001).

As flow cytometry data indicated a CMV-driven proliferation of CD8^+^*γδ* T cells that displayed effector phenotype and preferentially enriched with V*γ*9^−^*γδ* T cells, we, therefore, sought to characterize the TRG CDR3 clonotypes of *γδ* T cells to determine if there are differences regarding TCR diversity or clonal focusing driven by CMV infection. The CMV+ graft displayed several single clone expansions, as depicted by treemap (Figure 4(a)) and quantile plots (Figure 4(b)). Consequently, the TCR diversity was consistently lower in the CMV+ graft when compared to the CMV-, including inverse Simpson's D (85.19 vs. 302.50), Efron-Thisted estimator (5347.45 vs 57100.37), and iChao1 estimate (3090.92 versus 51054.66). Additionally, the CMV+ graft presented reduced singleton frequency (clones met once in the repertoire, 0.51% versus 39.00%, Figure 4(b)), high space taken by the top 10 most abundant clones (31.72% versus 11.34%, Figures 4(b) and 4(c)), high frequency of hyperexpanded clones (30.85% vs. 8.38%, Figure 4(d)), and high clonality (0.30 vs. 0.15), altogether demonstrating a high clonal focusing in this sample.

**Figure 5:**
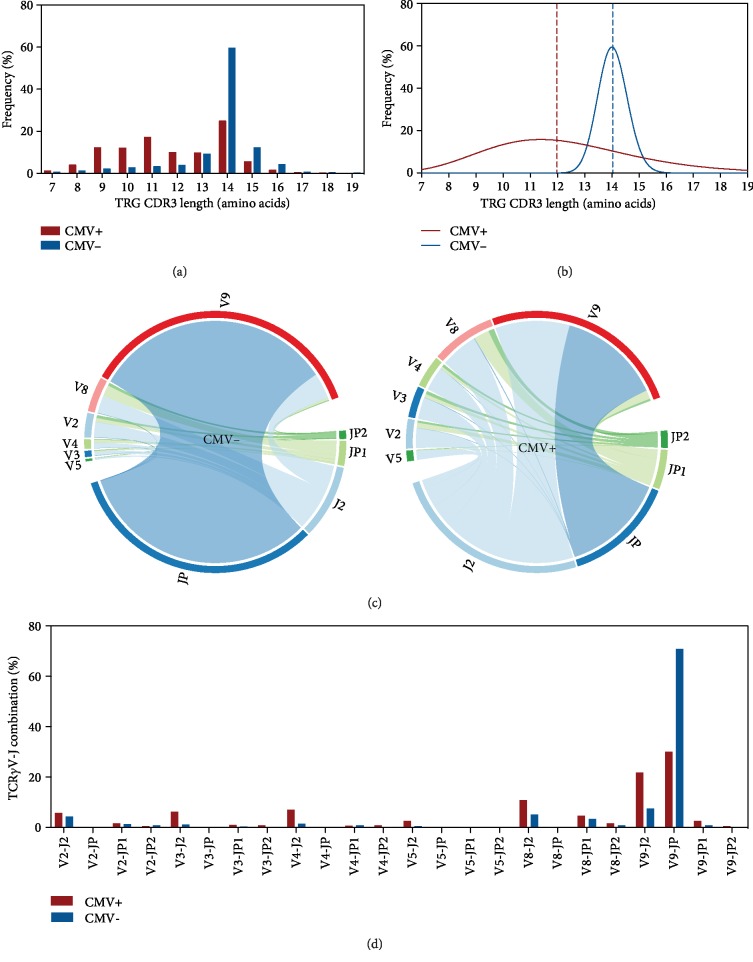
CMV positivity is associated with TRG reshaping and V-J segment usage changes. (a) TRG spectratype. Bars represent the frequency of unique CDR3 sequences with different amino acid lengths in a CMV+ and CMV- graft donor. (b) The distribution pattern of the clonotypes shown in (a). Lines represent the nonlinear curve fitting (Gauss function) in each donor. The vertical line indicates the median length in each donor. (c) V-J segment pairing abundance in CDR3 junctions of each donor based on CMV status. Chord diagrams are used for visualization. Ribbons connecting segment pairs are scaled by corresponding V-J pair frequency. (d) The frequency of different TRGV-TRGJ rearrangements shown in (c). Bars represent the usage of a given V-J junction in each graft.

Consistent with our previous work [19], the CMV- graft presented a high proportion of clones with a TRG consisting of 14 amino acids (59.44% vs. 24.81% in the CMV+ graft). In contrast, the CMV+ graft presented an enrichment of clones with a TRG length of 7 to 12 amino acids and a reduced frequency of those with 14 to 16 amino acid length (Figure 5(a)). These changes resulted in the shift from a Gaussian-distributed spectratype observed in the CMV- graft to a skewed repertoire in the CMV+ graft (Figure 5(b)). Furthermore, by evaluating the V-J pairing, we found that V*γ*9/JP segments were the most commonly used segments in the CMV- graft. In the CMV+ graft, the V*γ*3/J2, V*γ*4/J2, V*γ*5/J2, V*γ*8/J2, and V*γ*9/J2 were more used, while the V*γ*9-JP pair was dramatically reduced (Figures 5(c) and 5(d)).

As NGS data showed clonal focusing in CMV+ grafts, we hypothesized that CD8^+^*γδ* T cells are more clonally focused. To investigate this further, we assessed the TCR repertoire in sorted CD8^+^ and CD8^−^*γδ* T cells by CDR3 spectratyping. Analysis of two CMV+ grafts revealed a more focused TCR repertoire in CD8^+^*γδ* T cells compared to CD8^−^*γδ* T cells ([Supplementary-material supplementary-material-1]).

### 3.5. TCR/CD3 Stimulation Triggers CD8^+^*γδ* T Cells

**Figure 6:**
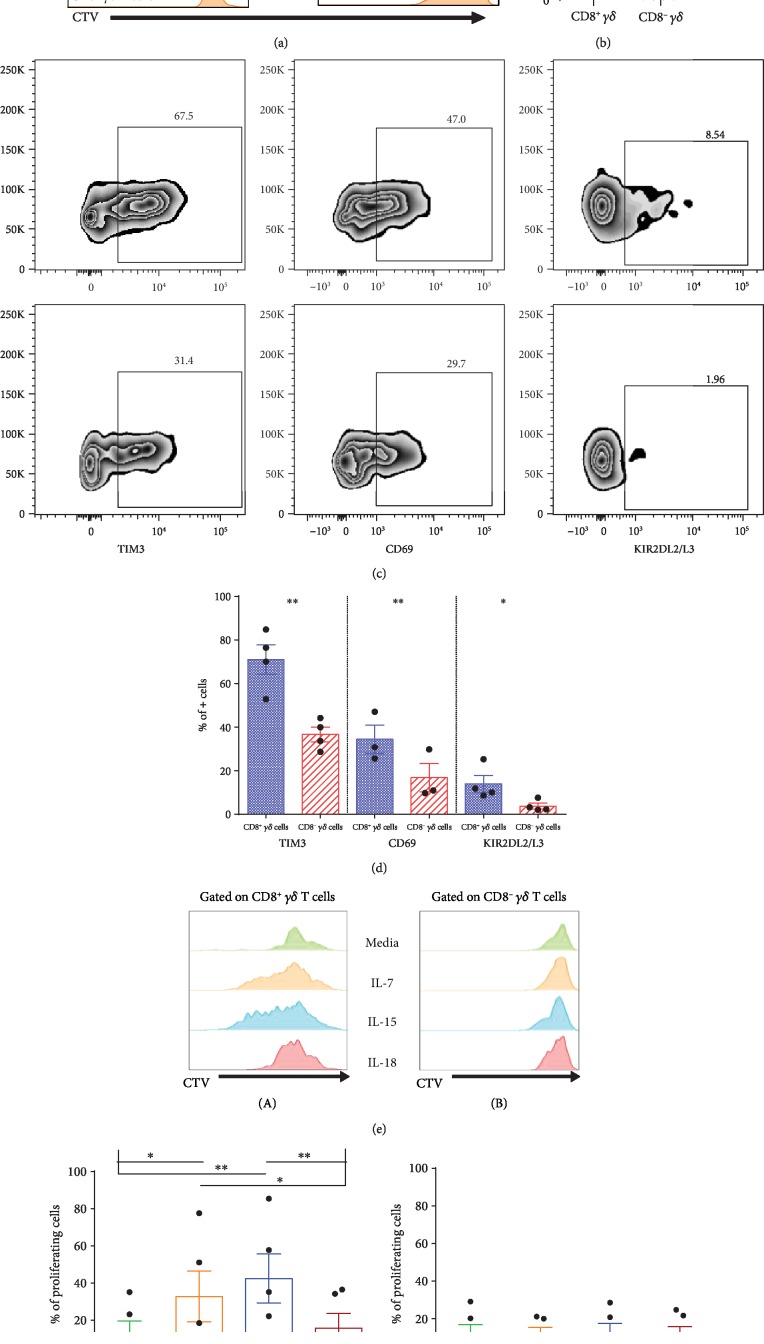
Enhanced response of CD8^+^*γδ* T cells to TCR and cytokine stimulation. (a) representative histograms (*n* = 4) of proliferating T cells from unstimulated or TCR/CD3 stimulated condition, gated on TCR *γδ*^−^ CD8^+^ (blue), CD8^+^*γδ* (green), and CD8^−^*γδ* (orange). (b) Proportions of proliferating CD8^+^*γδ* (green) and CD8^−^*γδ* (orange) cells after TCR/CD3 stimulation. (c) Representative FACS plot of TIM3, CD69, and KIR2DL2/L3 gated on CD8^+^*γδ* (upper) and CD8^−^*γδ* (lower) T cells after TCR/CD3 stimulation. (d) Proportions of CD8^+^*γδ* T cells (blue) and CD8^−^*γδ* T cells (red) expressing TIM3+, CD69+, and KIR2DL2/L3+ after TCR/CD3 stimulation. Paired *t*-test used. (e) Representative histogram of proliferation of CD8^+^*γδ* (A) and CD8^−^*γδ* (B) cultured in the presence of medium only (green), IL-7 (orange), IL-15 (blue), and IL-18 (red). Proportions of proliferating cells after culture in a medium, IL-7, IL-15, and IL-18 in CD8^+^*γδ* (f) and CD8^−^*γδ* (g). Repeated measures ANOVA is used. Bar and whiskers represent the mean and S.E.M.

As our results suggested an adaptive-like phenotype of CD8^+^*γδ* T cells, we sought to alleviate the potential role of TCR in the activation and proliferation of CD8^+^*γδ* T cells. TCR/CD3 stimulation resulted in significantly increased proliferation of CD8^+^*γδ* T cells compared to CD8^−^*γδ* T cells (Figures 6(a) and 6(b)). Furthermore, this TCR-driven proliferation was accompanied by increased frequencies of CD69^+^, TIM3+, and KIR2DL2/L3+ *γδ* T cells in CD8^+^*γδ* T cells compared to CD8^−^*γδ* T cells (Figure 6(c) and 6(d)) indicating their activation.

### 3.6. *γδ* T Cell Proliferation in response to Cytokine Stimulation

Next, we tested the impact of different cytokines on *γδ* T cell proliferation (Figure 6(e)). Interestingly, we observed a remarkably increased proliferation of CD8^+^*γδ* T cells in response to IL-7 and IL-15 but not to IL-18. In contrast, there was no significant difference in the proliferation of CD8^−^*γδ* T cells upon stimulation with IL-7, IL-15, or IL-18 (Figures 6(e)–6(g)).

## 4. Discussion

Consistent with previous reports, we showed higher proportions of V*δ*1^+^*γδ* T cells in CMV+ grafts. In fact, it has been shown that V*δ*1^+^*γδ* subset can pair to any V*γ* chains including V*γ*9 [13]. Of note, FACS data alone cannot show whether V*δ*1 couple to the semi-invariant or the noninvariant V*γ*9 chain. Using NGS, we showed less prevalence of J*γ*P-V*γ*9 pairing in CMV+ grafts [19]. In their study, Vermijlen et al. showed that CMV-responsive *γδ* T cells were restricted to V*γ*9^−^ subset, irrespective of the V*δ* chain usage [21]. Furthermore, a recent study showed that a distinct subset of V*δ*2^+^*γδ* T cells expresses V*γ*9^−^ (V*γ*9^−^V*δ*2^+^) and displayed an adaptive-like phenotype [22]. Therefore, V*γ*9^−^ subset of *γδ* T cells can better represent the adaptive-like compartment of *γδ* T cells compared to V*δ*2^neg^ subset. In this context, our results showed an increased frequency of V*γ*9^−^*γδ* T cells in BM grafts from CMV+ compared to CMV- donors (*P* = 0.05) and were preferentially differentiated to TE phenotype (CD27^low/-^CD45RO^−^) supporting an adaptive role of V*γ*9^−^*γδ* T cells.

*γδ* T cells expressing the CD8 coreceptor represent an unusual subpopulation of *γδ* T cells. Compared to the more common CD4^−^CD8^−^*γδ* T cells, their development and function are poorly understood. Reports have shown that CD8^+^*γδ* T cells selectively localize to intestinal epithelial tissue and are mostly V*δ*1^+^ [23, 24]. Furthermore, a potential role in intestinal inflammatory diseases has been recently described [15]. In line with previous report, we showed that CD8^+^*γδ* T cells are more frequent in CMV+ grafts [25] and express V*γ*9^−^*γδ* T cells. This CMV-driven proliferation was accompanied by a remarkable transition from CD27^+^ to CD27^low/-^ phenotype, indicating differentiation from naïve towards effector phenotype. Consistently, lymphoid homing receptor CCR7 in CD8^+^*γδ* T cells from CMV- grafts was higher as compared to CMV+ grafts, inferring their potential for homing to secondary lymphoid tissues and supporting a naïve phenotype in CMV- grafts. Whether this entails their ability to be primed by antigen-presenting cells in an adaptive-like manner remains to be investigated.

Importantly, it has been reported that CD8 coreceptors expressed by *γδ* T cells are mostly CD8*αα*, in contrast to CD8*αβ* expressed by conventional T cells [25]. In this regard, our study is limited as we have not assessed whether CD8*αα* or CD8*αβ* was mainly expressed; given the recent evidence on CD8*αβ*^+^*γδ* T cells [15], further characterization will be required to alleviate the immunobiological role of the different CD8 molecules.

We showed in a recent study that CD8^+^*γδ* T cells, in contrast to CD8^−^*γδ* T cells, displayed higher proliferation and activation markers in response to allogeneic stimulation [16]. Furthermore, their proportions in stem cell grafts were associated with the incidence of acute graft-versus-host disease (GVHD), supporting potential alloreactivity [16]. In the present study, we extended our findings by showing that CD8^+^*γδ* T cells were more responsive to TCR stimulation (Figure 6) and their response pattern to cytokines was different from CD8^−^*γδ* T cells, suggesting adaptive rather than innate response. Of note, the relationship between CMV reactivation and GVHD development after HCT is complex and can be bidirectional [26]. In light of our findings with regard to CD8^+^*γδ* T cells in stem cell grafts, it is valid to address whether CMV immune response and alloreactivity represent the dual face of this subset.

Consistent with previous reports, NGS results indicated repertoire perturbation in the form of TRG clonal focusing and higher usage of non-V*γ*9 gene segments in *γδ* T cells from the CMV+ graft. Of note, CMV+ grafts were enriched with CD8^+^*γδ* T cells that strongly displayed a terminal effector memory phenotype, indicating that clonal focusing revealed by NGS represents the clonal proliferation of CD8^+^*γδ* T cells. Though we have demonstrated in small scale spectratype that TCR repertoire was more clonally focused in CD8^+^*γδ* T cells, our study is limited as NGS analysis was not done on sorted CD8^+^*γδ* T cells to confirm this.

In conclusion, we showed that *γδ* T cell repertoire within BM grafts is reshaped by donor CMV serostatus and has provided evidence for the implication of CD8^+^*γδ* T cells in the HCMV immune response. Further studies are required to confirm our findings and to in-depth alleviate the impact of CMV-induced TCR repertoire and phenotypic changes of CD8^+^*γδ* T cells.

## Data Availability

The data used to support the findings of this study are available from the corresponding author upon request.

## Supplementary Materials

Supplementary Materials Table S1: sequences of primers used for spectratyping. V*γ* = variable gamma; C*γ* = constant gamma; C*δ* = constant delta.
