# Supplementary material for: CD8+γδ T Cells Are More Frequent in CMV Seropositive Bone Marrow Grafts and Display Phenotype of an Adaptive Immune Response
Source: Stem Cells Int. 2019 Dec 6;2019:6348060. doi: 10.1155/2019/6348060 (PMC6925825; doi:10.1155/2019/6348060)
Supplement: Supplementary Materials — Table S1: sequences of primers used for spectratyping. Vγ = variable gamma; Cγ = constant gamma; Cδ = constant delta. [file 6348060.f1.pdf]

Table S1 primers for Spectratyping

| primer | Sequence 5`-3` |
|---|---|
| Vγ2 | GCAAGCACAAGGAASAACTTGAG |
| Vγ3 | GTACTATGACGTCTCCACCG |
| Vγ4 | ATGACTCCTACACCTCCAGC |
| Vγ5 | CCCAGGAGGTGGAGCTGGAT |
| Vγ9 | ATCAACGCTGGCAGTCC |
| Vδ1 | CTGTCAACTTCAAGAAA-GCAGCGAAATC |
| Cγ-FAM | AATAGTGGGCTTGGGGGAAAC |
| Cδ-FAM | ACGGATGGTTTGGTATGAG GCTGA |

# Figure S1

A

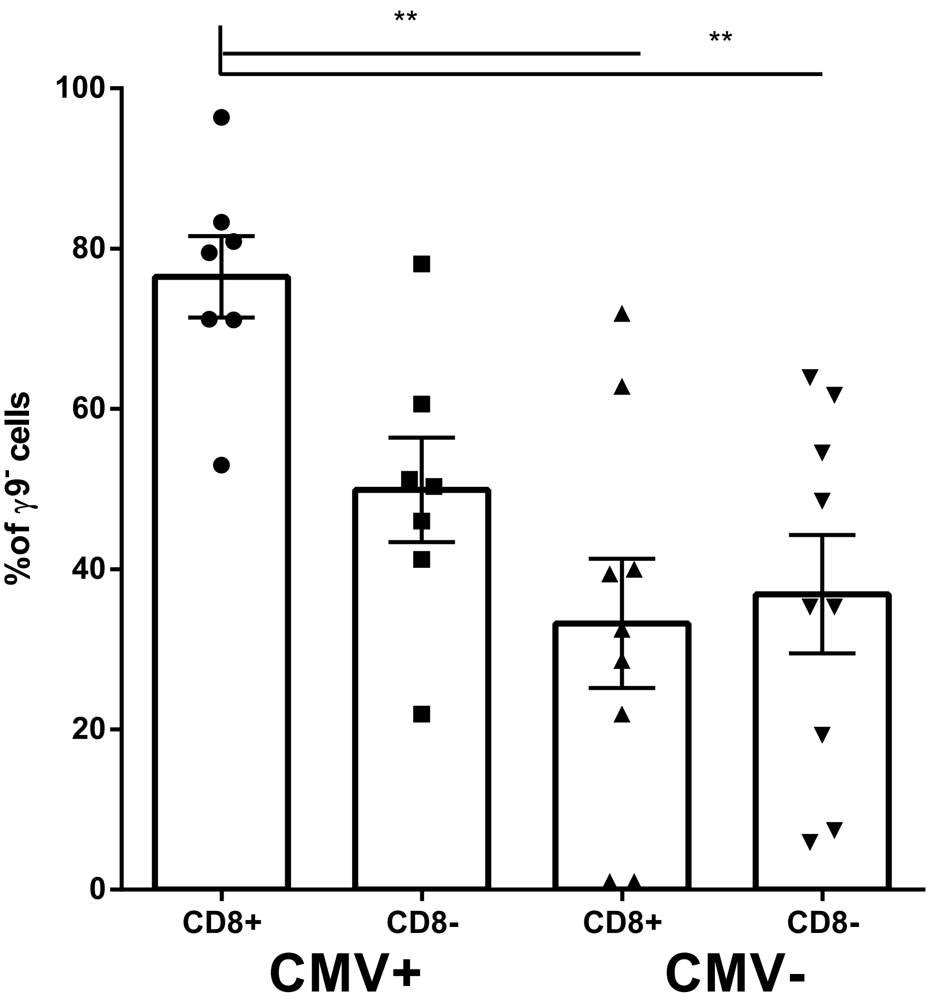

**
**
100
80
60
40
20
0
%of γ9- cells
CD8+
CD8-
CD8+
CD8-
CMV+
CMV-


B

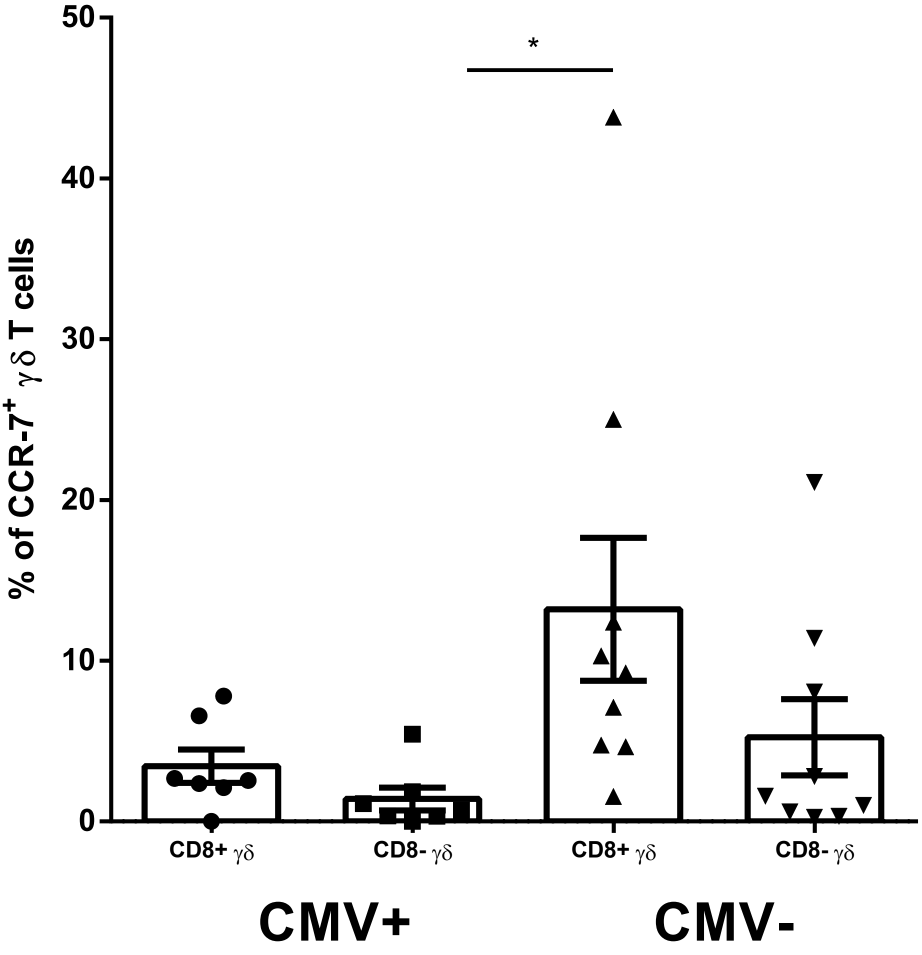

*
50
40
30
20
10
0
% of CCR-7+ γδ T cells
CD8+ γδ
CD8- γδ
CD8+ γδ
CD8- γδ
CMV+
CMV-

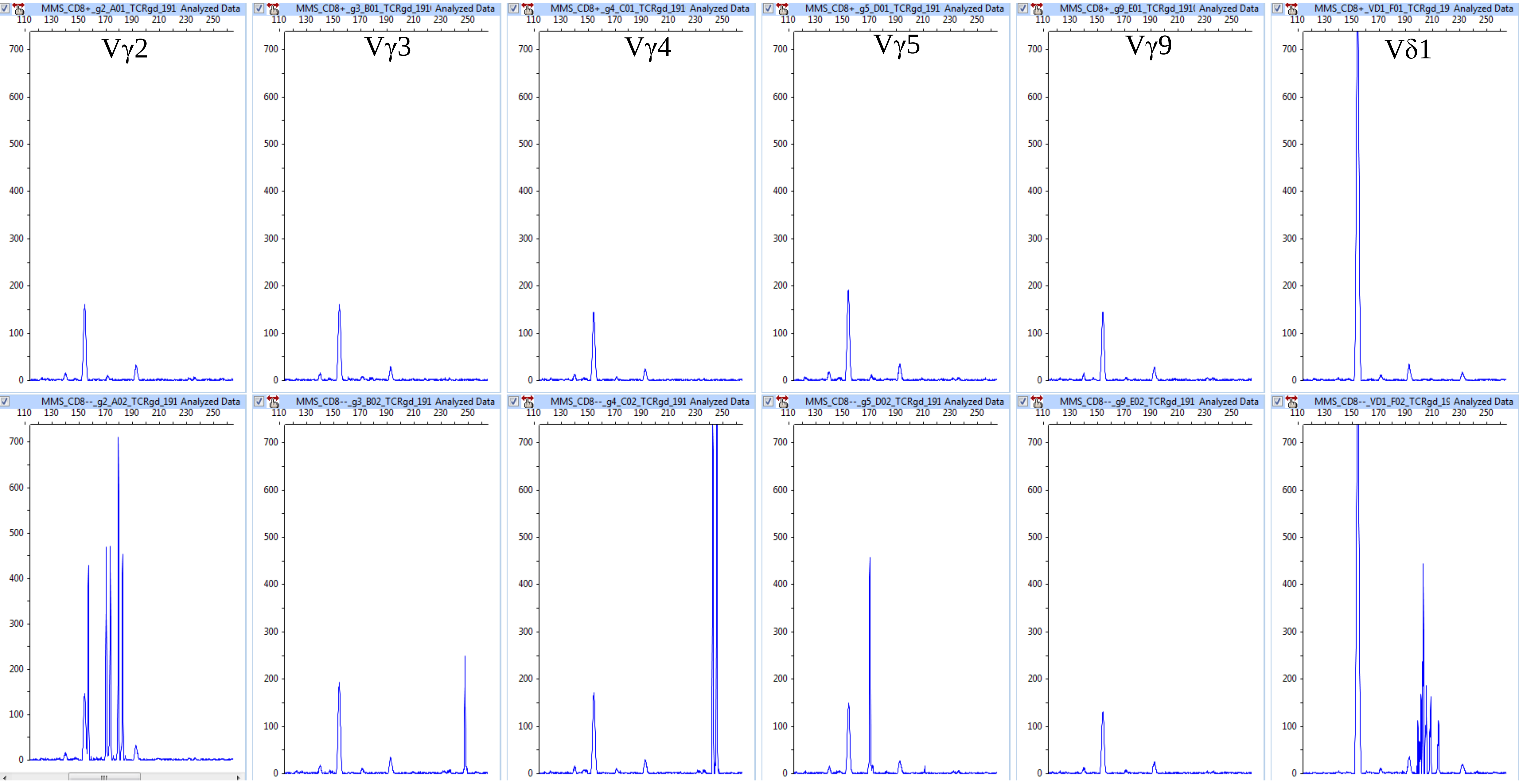
Vγ2
Vγ3
Vγ4
Vγ5
Vγ9
Vδ1
MMS_CD8+_g2_A01_TCRgd_191 Analyzed Data
MMS_CD8+_g3_B01_TCRgd_191 Analyzed Data
MMS_CD8+_g4_C01_TCRgd_191 Analyzed Data
MMS_CD8+_g5_D01_TCRgd_191 Analyzed Data
MMS_CD8+_g9_E01_TCRgd_191 Analyzed Data
MMS_CD8+_VD1_F01_TCRgd_19 Analyzed Data
MMS_CD8--_g2_A02_TCRgd_191 Analyzed Data
MMS_CD8--_g3_B02_TCRgd_191 Analyzed Data
MMS_CD8--_g4_C02_TCRgd_191 Analyzed Data
MMS_CD8--_g5_D02_TCRgd_191 Analyzed Data
MMS_CD8--_g9_E02_TCRgd_191 Analyzed Data
MMS_CD8--_VD1_F02_TCRgd_19 Analyzed Data
110 130 150 170 190 210 230 250
0 100 200 300 400 500 600 700
